# Trends in Publicly Funded Hospital Admissions and Reimbursement Associated With Non-melanoma Skin Cancer in Brazil, 2008-2023: A Nationwide Time-Series Study

**DOI:** 10.7759/cureus.115489

**Published:** 2026-08-31

**Authors:** Marcio Paulo C Salgueiro, Leticia P Scaramussa, Marcella S Abizaid, Sofia G Calil Lopes, Eduarda S Guedes, Maria Eduarda D Prudente, Mariana M Rizzolli, Chrystianne Ferrreira da Silva B Cunha, Tamires R Toqueton, Alana Leão

**Affiliations:** 1 Faculty of Medicine, Centro Universitário São Camilo, São Paulo, BRA; 2 Faculty of Medicine, Universidade do Grande Rio (UNIGRANRIO), Rio de Janeiro, BRA; 3 Faculty of Medicine, Universidade de Santo Amaro, São Paulo, BRA; 4 Faculty of Medicine, Universidade de Marília, Marília, BRA; 5 Faculty of Medicine, Anhembi Morumbi University, São Paulo, BRA; 6 Faculty of Medicine, Universidade de Caxias do Sul, Caxias do Sul, BRA; 7 Faculty of Medicine, Centro Universitário Alfredo Nasser (UNIFAN), Aparecida de Goiânia, BRA; 8 Faculty of Medicine, Universidade Evangélica de Goiás, Anápolis, BRA

**Keywords:** brazil, health expenditure, hospitalisation, keratinocyte carcinoma, non-melanoma skin cancer, skin neoplasms

## Abstract

Background

Non-melanoma skin cancer is common, but its health-system burden is incompletely represented by incidence and mortality estimates because most care is delivered outside conventional inpatient settings. We assessed long-term trends and regional variation in publicly financed hospital admissions and admission-related reimbursement associated with non-melanoma skin cancer in Brazil’s Unified Health System (SUS).

Methods

We conducted a nationwide ecological time-series study of admission authorizations recorded in the Hospital Information System of the Brazilian Unified Health System (SIH/SUS) from 2008 to 2023. SIH/SUS records reimbursed admission episodes rather than unique patients; readmissions, transfers, and multiple admissions for the same tumor cannot be identified. Admissions with a principal diagnosis coded as ICD-10 C44 were included as an operational measure of hospital care associated with non-melanoma malignant skin neoplasms and were classified according to the place of hospitalization. Annual crude and age-standardized admission rates were calculated for Brazil and its five macro-regions; direct standardization used the age distribution of the 2022 Brazilian Census. Approved SUS reimbursement was adjusted to 2023 Brazilian reais using the national consumer price index. Temporal trends in log-transformed admission counts, rates, total reimbursement, reimbursement per admission, and mean length of stay were estimated using Prais-Winsten regression and reported as annual percentage changes (APCs) with 95% CIs.

Results

Between 2008 and 2023, 535,750 admission episodes were recorded. Annual admissions increased from 13,974 to 60,075 (APC 9.44%, 95% CI 7.43 to 11.50), and the age-standardized rate increased from 10.4 to 28.0 per 100,000 population (APC 6.07%, 95% CI 4.10 to 8.07). Rates increased in the North, Southeast, South, and Central-West, whereas the Northeast showed no evidence of a temporal trend. Cumulative inflation-adjusted approved reimbursement was R$949.3 million, and annual reimbursement increased by 6.92% (95% CI 4.96 to 8.91). Reimbursement per admission decreased by 2.22% annually (95% CI -2.80 to -1.64), and mean length of stay decreased by 6.77% annually (95% CI -8.47 to -5.04). In 2023, the age-standardized rate ranged from 9.2 per 100,000 population in the North to 57.4 per 100,000 population in the South.

Conclusions

Publicly financed hospital admissions and total approved SUS reimbursement associated with ICD-10 C44 increased substantially in Brazil between 2008 and 2023, with marked regional variation. The increase in recorded reimbursement occurred alongside substantial growth in admission volume, despite declining reimbursement per episode and shorter recorded stays. These findings quantify the publicly financed hospital component of service use and should not be interpreted as population incidence, the number of unique patients, or total treatment costs.

## Introduction

Non-melanoma skin cancer, principally basal cell carcinoma and cutaneous squamous cell carcinoma, is among the most frequently diagnosed malignancies worldwide. Its global burden has increased over recent decades, particularly among older populations [1,2]. Quantification remains difficult because many lesions are managed in ambulatory settings, and keratinocyte cancers are incompletely captured by population-based cancer registries [3].

Low disease-specific mortality can obscure the demands that non-melanoma skin cancer places on health services. The great majority of lesions are excised in outpatient or day-surgery settings and never generate a hospital admission authorisation. A minority require hospital-based management and therefore enter the SIH/SUS records. These include extensive or recurrent tumours requiring wide excision, lesions at anatomically complex sites where reconstruction with flaps or grafts is needed, tumours requiring general or regional anaesthesia, and cases involving patients whose immunosuppression, multimorbidity, or advanced age makes outpatient management unsuitable. Admissions recorded in SIH/SUS therefore reflect care delivered to a selected, more complex subset of the treated population. National administrative studies in Portugal and Germany found that the aggregate hospital burden of non-melanoma skin cancer exceeded that of melanoma because of the substantially greater volume of care [4,5]. Treatment expenditure for skin cancer has also increased in the United States [6].

Evidence from Brazil is limited. A study from São Paulo estimated that aggregate expenditure for non-melanoma skin cancer in the public system exceeded that for melanoma, despite a lower cost per treated case [7]. A more recent national analysis reported increasing hospital morbidity from malignant skin neoplasms and substantial geographic variation but combined melanoma and non-melanoma diagnoses [8]. No Brazilian study has jointly examined age-standardised C44 admission rates, inflation-adjusted reimbursement, and length of stay over a long national time series.

The Brazilian Hospital Information System provides nationwide information on hospital admission episodes financed by the Unified Health System and can therefore quantify recorded hospital activity and its associated reimbursement rather than the occurrence of disease in the population. Our primary objective was to estimate the temporal trend in the age-standardised rate of hospital admissions associated with ICD-10 C44 in Brazil between 2008 and 2023. Our secondary objectives were to estimate corresponding trends in admission counts, crude rates, total approved SUS reimbursement, reimbursement per admission, and mean length of stay and to characterise variation in these trends across the five macro-regions.

## Materials and methods

Study design and reporting

We conducted a nationwide ecological time-series study using routinely collected, publicly available, aggregated data for the period from January 1, 2008 to December 31, 2023. The units of analysis were annual population-level observations for Brazil and its five macro-regions: North, Northeast, Southeast, South, and Central-West. The national series was extracted independently from the SIH/SUS TabNet platform rather than derived by aggregating the five regional series; the two were confirmed to be numerically consistent. Reporting followed the Strengthening the Reporting of Observational Studies in Epidemiology (STROBE) and REporting of studies Conducted using Observational Routinely-collected health Data (RECORD) statements [9,10].

Data sources and study population

Hospital data were obtained from the Hospital Information System of the Brazilian Unified Health System (SIH/SUS), accessed through the DATASUS TabNet platform (module Morbidade Hospitalar do SUS, by place of hospitalisation) on 7 July 2026 [11]. SIH/SUS is derived from hospital admission authorisations (Autorização de Internação Hospitalar, AIH) submitted for reimbursement by public hospitals and private or philanthropic institutions contracted by the Unified Health System. Only AIHs that are approved and paid are included in this module, which is the standard basis for SIH/SUS morbidity tabulations.

We included admissions with a principal diagnosis coded as C44 according to the International Classification of Diseases, 10th Revision (ICD-10). C44 was used operationally to identify hospital admissions associated with non-melanoma malignant skin neoplasms. Throughout this manuscript, findings are described in terms of admissions with a principal diagnosis of ICD-10 C44 rather than as confirmed cases of non-melanoma skin cancer because SIH/SUS does not provide histopathological confirmation and cannot reliably distinguish basal cell carcinoma, cutaneous squamous cell carcinoma, or less common malignant skin tumours. Admissions coded as melanoma of the skin (C43) were excluded.

Data were extracted by macro-region, sex, and the SIH/SUS quinquennial age-group classification (18 categories, from under 1 year to 80 years and over); for direct age standardisation, the two youngest categories were collapsed into a single 0-4 years group, yielding 17 age strata. Age and geographic information are mandatory fields for AIH authorisation, and no admissions were assigned to an unknown-age or unknown-geography category in any extracted table.

Because SIH/SUS records admission episodes rather than unique individuals, readmissions could not be identified, and the publicly tabulated data used in this study do not permit the identification or deduplication of interhospital transfers, supplementary AIHs, or multiple admission authorisations relating to the same individual or tumour. This is an inherent limitation of aggregated SIH/SUS data, shared by the great majority of published ecological analyses using this source, rather than a limitation specific to this study.

Population denominators and standard population

Annual population denominators were obtained from the Brazilian Institute of Geography and Statistics (IBGE) population projections for 2010-2060, with retrojected estimates for 1996-2009, produced by sex and age group [12]; this is the denominator series adopted by DATASUS for the calculation of health indicators. The age distribution of the Brazilian population enumerated in the 2022 Demographic Census (IBGE) [13] was used as the standard population for direct age standardisation and was applied uniformly across all years and macro-regions to ensure regional comparability. The full set of standard population weights by five-year age group is provided in Appendix 1.

Outcomes

The primary outcome was the annual age-standardised hospital admission rate associated with ICD-10 C44, expressed per 100,000 population. Secondary outcomes were the annual number of admissions, crude admission rate, total SIH/SUS reimbursement, reimbursement per admission, and mean length of stay. Absolute counts and population-based rates were reported together because changes in admission volume can reflect population growth or population ageing even when rates remain unchanged.

Crude rates were calculated as the number of annual admissions divided by the corresponding population and multiplied by 100,000. Age-specific rates were calculated for each calendar year and macro-region and were standardised using the direct method and the age distribution of the 2022 Brazilian Census. Monetary values were adjusted using the annual average of the Extended National Consumer Price Index (Índice Nacional de Preços ao Consumidor Amplo; IPCA) and expressed in 2023 Brazilian reais [14]. Reimbursement per admission was calculated as the total annual reimbursement divided by the total annual number of admissions for the corresponding region-year.

Mean length of stay was obtained directly from the SIH/SUS Média de permanência field rather than derived. In SIH/SUS, length of stay is computed as the discharge date minus the admission date; therefore, an admission and discharge occurring on the same day contributes zero days. A mean length of stay approaching one day may therefore reflect an increasing proportion of same-day or short-stay procedural admissions rather than a data artefact, a pattern consistent with the increasing use of ambulatory and short-stay management for this condition.

Statistical analysis

Temporal trends were estimated using Prais-Winsten generalised least-squares regression to account for first-order serial autocorrelation in consecutive annual observations. The dependent variable was the base-10 logarithm of each annual indicator, and calendar year was the independent variable. Separate models were fitted for Brazil and each macro-region for annual admission counts, age-standardised admission rates, inflation-adjusted total reimbursement, inflation-adjusted reimbursement per admission, and mean length of stay.

The annual percentage change (APC) was calculated from the regression coefficient according to the expression APC = (10^β - 1) × 100, where β represents the coefficient for calendar year. The corresponding 95% CIs were calculated by transforming the lower and upper limits of the regression coefficient. A trend was classified as increasing when the APC was positive and its 95% CI excluded zero, decreasing when the APC was negative and its CI excluded zero, and stable when the CI included zero.

Regional differences in temporal trends were evaluated using a pooled model containing calendar year, macro-region, and year-by-region interaction terms, with the statistical significance of regional heterogeneity assessed using a joint test of the interaction terms. This model assumed that observations across years within each region were independent and did not incorporate a region-specific autocorrelation structure. Therefore, the per-region trend estimates, each independently corrected for first-order autocorrelation, constitute the primary basis for regional comparison, whereas the pooled interaction test is presented as a descriptive summary of heterogeneity.

Residual serial correlation was assessed using the autocorrelation coefficient (ρ) and the Durbin-Watson statistic for each fitted series, together with graphical residual diagnostics; the full results are reported in Appendix 2. Sensitivity analyses repeated the primary trend models (i) using crude rather than age-standardised rates and (ii) excluding the 2020-2021 pandemic period to assess the robustness of the estimated trends to population ageing and the acute disruption of health-service delivery during the COVID-19 pandemic, respectively. Because regional and outcome-specific trend estimates were compared descriptively across multiple strata without formal adjustment for multiplicity, these comparisons should be interpreted as exploratory.

All tests were two-sided, and p < 0.05 was considered statistically significant. Analyses were performed using Python (version 3.13), with custom implementations of the Prais-Winsten estimator and direct age standardisation. Before application to the study data, the Prais-Winsten estimator was validated against synthetic time series with an exactly specified trend, for example, a series constructed with a precisely defined annual growth rate of 5.00%, which the estimator recovered to four decimal places. The analytical code has been archived and is available on request. No joinpoint, data-driven breakpoint, or interrupted time-series analysis was performed. The analysis estimated the average long-term trend across the complete study period.

Ethical considerations

The study used only aggregated, publicly available data, and no individual-level or identifiable data were accessed at any stage of the analysis. Under Brazilian regulations, studies using public-domain information or databases containing only aggregated information without the possibility of individual identification are not registered or evaluated within the national CEP/CONEP system, pursuant to Resolution No. 510/2016 of the National Health Council (Conselho Nacional de Saúde) [15].

## Results

National admission volume and trend

Between 2008 and 2023, 535,750 hospital admissions associated with ICD-10 C44 were recorded in SIH/SUS. Annual admissions increased from 13,974 in 2008 to 60,075 in 2023, corresponding to an APC in the annual admission count of 9.44% (95% CI: 7.43 to 11.50; p < 0.001), substantiating that growth in service volume was a principal driver of the observed trends. The crude admission rate increased from 7.4 to 27.8 per 100,000 population, with an APC of 8.50% (95% CI 6.54 to 10.48; p < 0.001). The age-standardised admission rate increased from 10.4 to 28.0 per 100,000 population, corresponding to an APC of 6.07% (95% CI 4.10 to 8.07; p < 0.001; Table 1 and Figure 1). The complete annual series of admission counts, crude rates, and age-standardised rates for Brazil is provided in Appendix 2.

**Table 1:** Temporal trends in age-standardized hospital admission rates associated with ICD-10 code C44 in Brazil and its macro-regions, 2008-2023. Data include total admission counts for 2008-2023, APCs, and 95% CIs. APCs and 95% CIs were estimated using Prais-Winsten regression of log10-transformed annual rates. Rates were directly age-standardized to the age distribution of the 2022 Brazilian Census (Appendix 1) and are expressed per 100,000 population. APC: Annual percentage change; ICD-10: International Classification of Diseases, 10th Revision.

Region	Admissions (n)	APC, %	95% CI	p-value	Trend classification
Brazil	535,750	6.07	4.10 to 8.07	< 0.001	Increasing
North	9,692	6.49	3.60 to 9.46	< 0.001	Increasing
Northeast	96,322	2.05	-2.81 to 7.17	0.387	No evidence of a log-linear trend
Southeast	216,796	6.45	2.94 to 10.09	0.001	Increasing
South	172,594	7.15	5.19 to 9.14	< 0.001	Increasing
Central-West	40,346	4	2.37 to 5.64	< 0.001	Increasing

**Figure 1:**
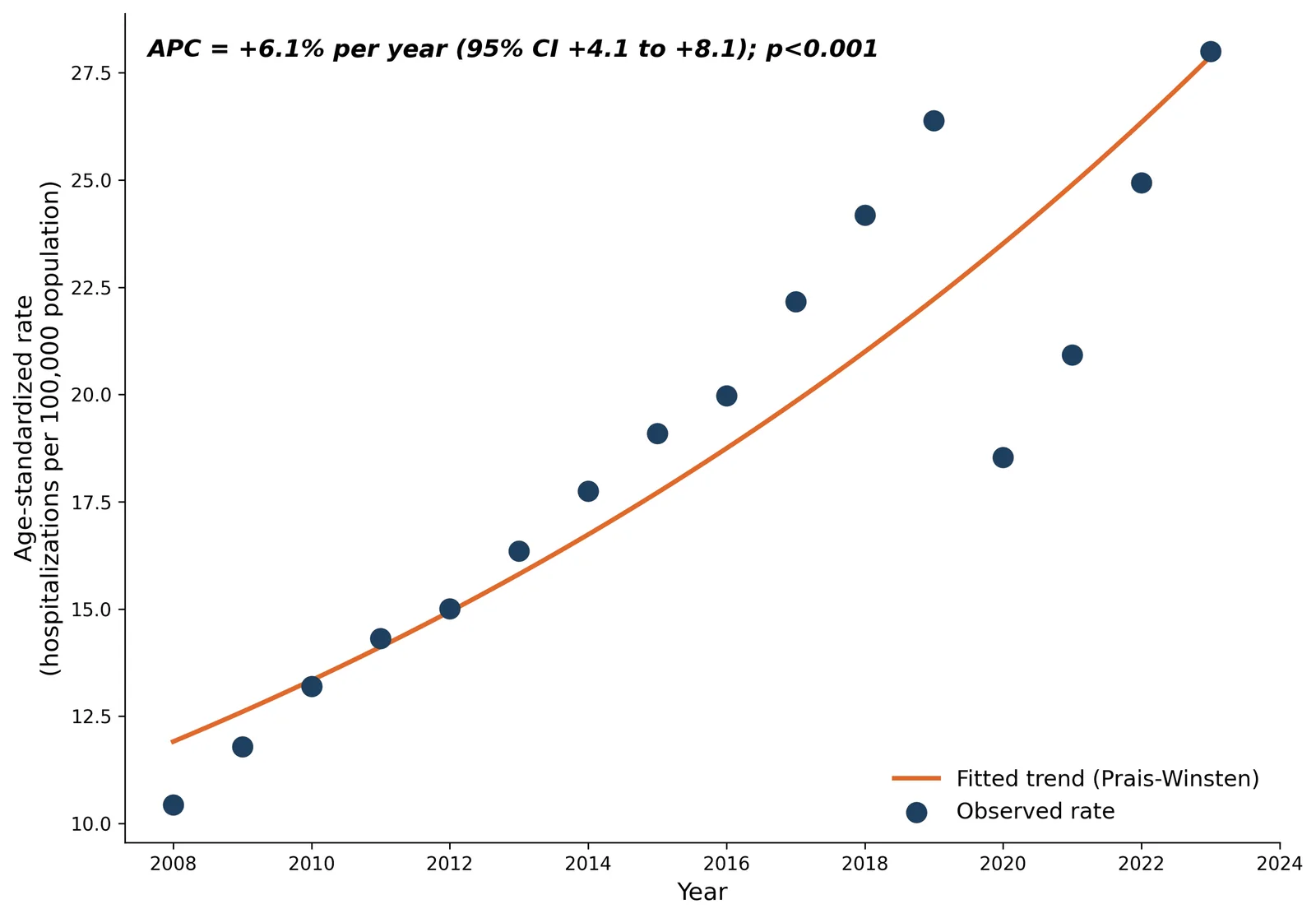
National trend in hospitalisation for non-melanoma skin cancer in Brazil, 2008-2023. Dots show the observed annual age-standardized rates, and the solid line shows the fitted log-linear trend estimated using Prais-Winsten regression. Rates were standardized using the direct method to the age distribution of the 2022 Brazilian Census and are expressed as admissions per 100,000 population. The APC value is shown rounded to one decimal place; the exact estimate with its 95% CI is reported in Table 1. APC: Annual percentage change; ICD-10: International Classification of Diseases, 10th Revision.

Annual age-standardised rate were treated as complete administrative tabulations of approved SIH/SUS admissions rather than as estimates from a probability sample, and the corresponding crude and age-standardised rates were calculated using population denominators. Accordingly, no confidence intervals were computed for individual annual rates. Uncertainty in the estimated temporal trend was instead summarised using the 95% CI for the APC.

The age-standardised rate decreased from 26.39 per 100,000 population in 2019 to 18.53 per 100,000 population in 2020, a descriptive reduction of 29.8%. This percentage change was calculated directly from the observed annual rates and was not estimated using an interrupted time-series or breakpoint model. The rate subsequently recovered to the highest value in the series in 2023. The autocorrelation coefficient (ρ) and Durbin-Watson statistic for all outcomes, nationally and by region, are reported in Appendix 3.

Regional trends

Age-standardised admission rates increased in the North (APC 6.50%, 95% CI 3.59 to 9.49; Figure 2A), Southeast (APC 6.44%, 95% CI 2.93 to 10.08; Figure 2C), South (APC 7.14%, 95% CI 5.19 to 9.13; Figure 2D), and Central-West (APC 3.96%, 95% CI 2.35 to 5.58; Figure 2E). The Northeast showed no evidence of a log-linear trend (APC 2.03%, 95% CI -2.85 to 7.14; Table 1 and Figure 2B). In 2023, the highest age-standardised rate was observed in the South (57.4 per 100,000 population), and the lowest was observed in the North (9.2 per 100,000 population). The comparatively low rate in the North should not necessarily be interpreted as reflecting lower disease occurrence because it may instead reflect regional differences in healthcare access, service coverage, diagnostic coding practices, or the availability of different care settings. A pooled model containing calendar year, macro-region, and year-by-region interaction terms indicated regional heterogeneity (F(4,70) = 2.70; p = 0.037). This was a single, prespecified omnibus test of heterogeneity and was not subject to correction for multiple comparisons. As described in the Methods, the pooled test did not account for region-specific autocorrelation and was therefore treated as descriptive. By contrast, the descriptive regional and outcome-specific comparisons presented throughout the Results section span multiple strata without such correction and should be interpreted as exploratory. Regional admission counts alongside age-standardised rates are provided in Table 1.

**Figure 2:**
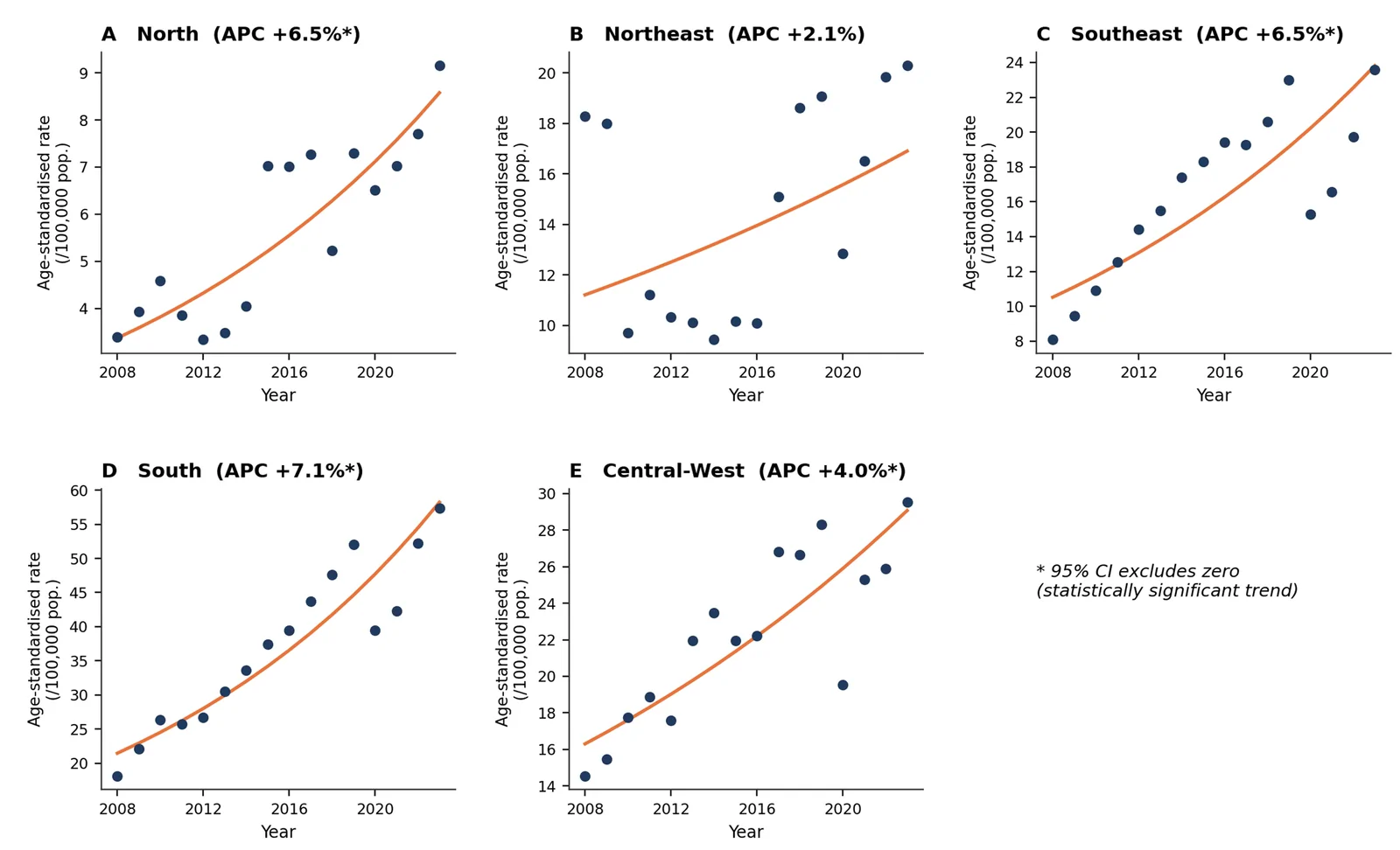
Regional trends in hospitalisation for non-melanoma skin cancer by macro-region in Brazil, 2008-2023. Dots show observed annual rates, and solid lines show region-specific fitted log-linear trends estimated by Prais-Winsten regression. Rates were standardised by the direct method to the age distribution of the 2022 Brazilian Census and are expressed as admissions per 100,000 population. Asterisks indicate APC estimates whose 95% CIs exclude zero. Panels: (A) North; (B) Northeast; (C) Southeast; (D) South; and (E) Central-West. APC values in the panel titles are shown rounded to one decimal place; exact estimates with 95% CIs are reported in Table 1. APC indicates annual percentage change.

Hospital reimbursement

Cumulative inflation-adjusted reimbursement was R$949.3 million in 2023 values. Annual reimbursement increased from R$31.6 million in 2008 to R$90.7 million in 2023, corresponding to an APC of 6.92% (95% CI 4.96 to 8.91; p < 0.001). Reimbursement increased in the South, Northeast, and Southeast; the North and Central-West showed no evidence of a log-linear trend (Table 2).

**Table 2:** Total approved SIH/SUS reimbursement associated with ICD-10 C44 admissions, adjusted for inflation to 2023 Brazilian reais, by macro-region in Brazil, 2008-2023. The annual percentage change (APC) was estimated using Prais-Winsten generalised least-squares regression of the base-10 logarithm of the annual indicator on calendar year, correcting for first-order serial autocorrelation. APC = (10^β - 1) × 100, where β represents the estimated coefficient for calendar year. A trend was classified as increasing or decreasing, according to the direction of the APC, when its 95% CI excluded zero and as showing no evidence of a log-linear trend when the CI included zero. Values shown for 2008 and 2023 are observed annual figures, not model-fitted values. ICD-10: International Classification of Diseases, 10th Revision; SIH/SUS: Hospital Information System of the Brazilian Unified Health System.

Region	2008 (R$)	2023 (R$)	APC (%)	95% CI	p-value	Trend classification
Brazil	31,638,872.50	90,657,940.52	6.92	+4.96 to +8.91	< 0.001	Increasing
North	522,288.67	1,166,923.15	4.74	-0.05 to +9.75	0.052	No evidence of a log-linear trend
Northeast	11,739,897.65	32,240,395.12	7.29	+3.47 to +11.25	< 0.001	Increasing
Southeast	9,507,781.67	26,986,833.71	6.4	+3.22 to +9.68	< 0.001	Increasing
South	6,961,676.57	24,913,306.57	8.79	+6.12 to +11.52	< 0.001	Increasing
Central-West	2,907,227.93	5,350,481.97	3.95	-0.52 to +8.63	0.08	No evidence of a log-linear trend

Total reimbursement increased at a materially slower rate than admission volume (APC 9.44%) because reimbursement per admission declined over the same period (APC -2.22%; see below). The two trends combine approximately multiplicatively: (1 + 0.0944) × (1 - 0.0222) - 1 ≈ 7.0%, closely matching the observed APC in total reimbursement (6.92%) and indicating that the increase in total reimbursement was driven predominantly by rising admission volume and partially offset by declining reimbursement per admission.

Inflation-adjusted reimbursement per admission decreased from R$2,264.12 in 2008 to R$1,509.08 in 2023 (APC -2.22%, 95% CI -2.80 to -1.64; p < 0.001). Decreasing trends were observed in the North, Southeast, South, and Central-West, whereas the Northeast showed no evidence of a log-linear trend (Table 3 and Figure 3).

**Table 3:** Hospital reimbursement per admission associated with ICD-10 C44, adjusted for inflation to 2023 Brazilian reais, by macro-region in Brazil, 2008-2023. Annual percentage change (APC) was estimated by Prais-Winsten generalised least-squares regression on the base-10 logarithm of the annual indicator, with calendar year as the independent variable, correcting for first-order serial autocorrelation. APC = (10^β - 1) × 100, where β represents the estimated coefficient for calendar year. A trend was classified as increasing or decreasing when the 95% CI of the APC excluded zero and as showing no evidence of a log-linear trend when the CI included zero. Values shown for 2008 and 2023 are observed annual figures, not model-fitted values.

Region	2008 (R$)	2023 (R$)	APC (%)	95% CI	p-value	Trend
Brazil	2,264.12	1,509.08	-2.22	-2.80 to -1.64	< 0.001	Decreasing
North	1,852.09	1,032.68	-4.21	-7.24 to -1.08	0.012	Decreasing
Northeast	3,270.17	3,016.22	-0.57	-1.90 to +0.77	0.374	No evidence of a log-linear trend
Southeast	1,928.17	1,135.05	-3.42	-4.10 to -2.72	< 0.001	Decreasing
South	1,749.16	1,234.19	-1.75	-2.83 to -0.66	0.004	Decreasing
Central-West	2,441.00	1,246.04	-4.02	-7.02 to -0.93	0.015	Decreasing

**Figure 3:**
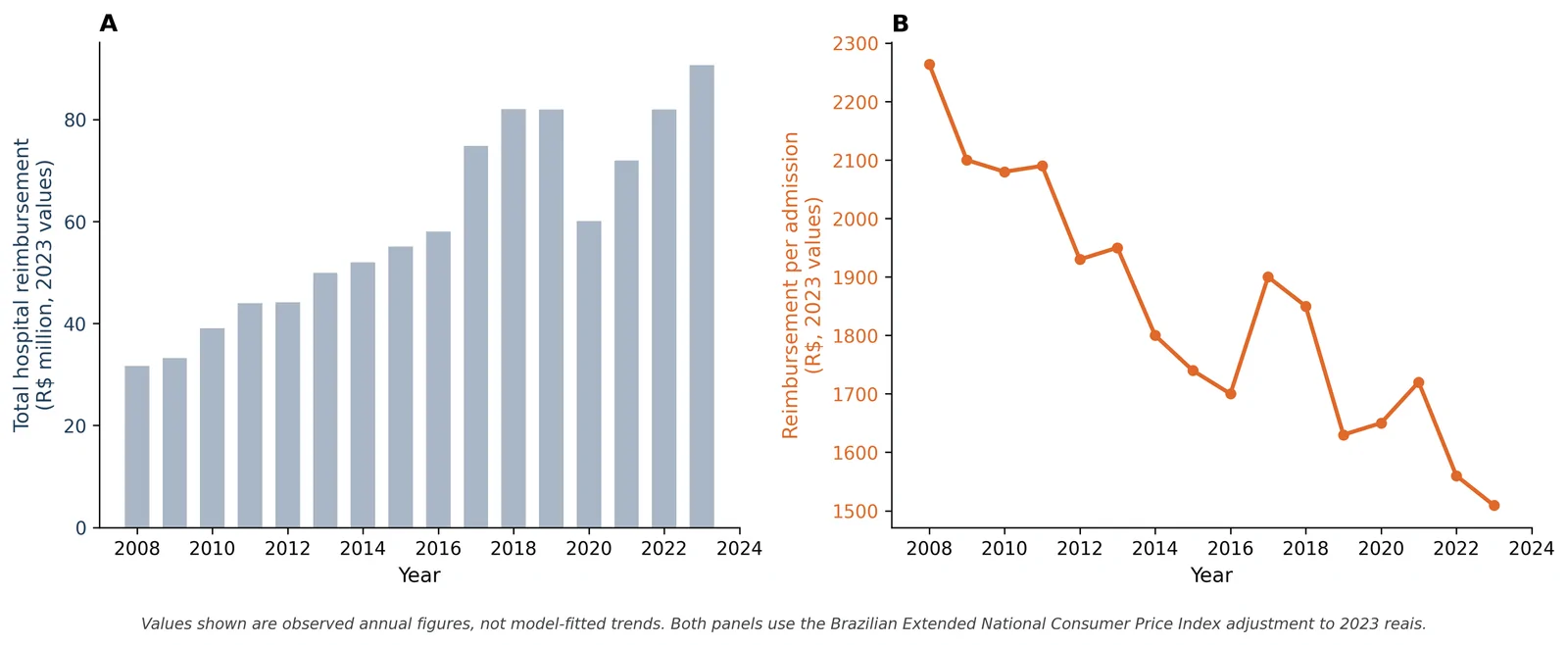
Total annual hospital reimbursement and reimbursement per admission associated with ICD-10 C44 admissions in Brazil, 2008-2023. (A) Total annual reimbursement, in millions of 2023 Brazilian reais. (B) Reimbursement per admission, in 2023 Brazilian reais. Values shown are observed annual figures, not model-fitted trends. Monetary values in both panels were adjusted using the Brazilian Extended National Consumer Price Index. ICD-10: International Classification of Diseases, 10th Revision.

Length of stay

Mean length of stay decreased from 2.76 days in 2008 to 0.97 days in 2023 (APC -6.77%, 95% CI -8.47 to -5.04; p < 0.001). Decreasing trends were observed in the Northeast, Southeast, South, and Central-West; the North showed no evidence of a single log-linear trend (Table 4). As detailed in the Methods, length of stay in SIH/SUS is computed as the discharge date minus the admission date; therefore, an admission and discharge occurring on the same calendar day contributes zero days. A national mean approaching one day may be consistent with an increasing contribution of same-day and short-stay procedural admissions toward the end of the study period. However, the aggregated TabNet tabulation provides only the annual mean length of stay by region and does not permit extraction of the underlying distribution or the proportion of same-day (zero-day) episodes. Individual-level microdata would be required to report this distribution directly, and we did not interpret the observed decline as evidence of improved clinical efficiency in the absence of procedure-level or clinical information.

**Table 4:** Mean length of hospital stay for ICD-10 C44 admissions by macro-region in Brazil, 2008-2023. Annual percentage change (APC) was estimated by Prais-Winsten generalised least-squares regression on the base-10 logarithm of the annual indicator, with calendar year as the independent variable, correcting for first-order serial autocorrelation. APC = (10^β - 1) × 100. A trend was classified as increasing or decreasing when the 95% CI of the APC excluded zero and as showing no evidence of a log-linear trend when the CI included zero. Values shown for 2008 and 2023 are observed annual figures, not model-fitted values. † In the North, the series was non-monotonic, rising to a peak of 9.4 days in 2016 before declining; “no evidence of a single log-linear trend” reflects the absence of a single log-linear pattern across the full period, not the absence of change.

Region	2008 (days)	2023 (days)	APC (%)	95% CI	p-value	Trend
Brazil	2.8	1	-6.77	-8.47 to -5.04	< 0.001	Decreasing
North	5.9	3	-4.66	-9.65 to +0.60	0.078	No evidence of a single log-linear trend†
Northeast	3.5	1.5	-5.53	-7.27 to -3.75	< 0.001	Decreasing
Southeast	2.9	0.9	-7.51	-10.02 to -4.93	< 0.001	Decreasing
South	1.8	0.6	-7.18	-8.84 to -5.49	< 0.001	Decreasing
Central-West	2.4	1.1	-5.5	-6.58 to -4.41	< 0.001	Decreasing

In the North, the series was non-monotonic, increasing from 5.9 days in 2008 to a peak of 9.4 days in 2016 before decreasing to 3.0 days in 2023. “No evidence of a single log-linear trend” reflects this non-monotonic pattern rather than an absence of change over the study period.

Sensitivity analyses

Sensitivity analyses were conducted using crude rather than age-standardised rates and by excluding the 2020-2021 pandemic period for Brazil and each macro-region (Table 5). The crude admission rate showed a steeper increase than the age-standardised rate in every geographic unit, consistent with the contribution of population ageing to the crude trend. After excluding 2020-2021, the direction and statistical significance of the age-standardised rate trend were preserved for Brazil and four of the five macro-regions; in the Northeast, the trend remained non-significant under both specifications.

**Table 5:** Sensitivity analyses: crude-rate and pandemic-period-excluded estimates of the annual percentage change in hospital admission rates associated with ICD-10 C44 in Brazil and its macro-regions, 2008-2023. The crude rate was calculated as annual admissions divided by the total population and multiplied by 100,000. “Excluding 2020-2021” refers to the age-standardised rate trend re-estimated with the two pandemic years removed from the series. APCs and 95% CIs were estimated by Prais-Winsten regression, as described in Table 1.

Region	Crude-rate APC (%)	95% CI	Age-standardised rate APC excluding 2020-2021 (%)	95% CI
Brazil	+8.50	+6.54 to +10.48	+6.56	+5.19 to +7.95
North	+7.69	+3.93 to +11.60	+6.74	+3.41 to +10.17
Northeast	+3.61	-1.61 to +9.10	+1.78	-4.07 to +7.99
Southeast	+9.16	+5.59 to +12.84	+6.43	+3.65 to +9.28
South	+9.80	+7.86 to +11.78	+7.62	+6.16 to +9.10
Central-West	+6.56	+5.04 to +8.10	+4.56	+3.26 to +5.88

## Discussion

In this nationwide analysis of more than half a million hospital admissions over a 16-year period, the age-standardized admission rate associated with ICD-10 C44 rose substantially, and the annual admission count increased more steeply than the standardized rate, indicating that growth in recorded hospital activity funded by SUS outpaced what population change and aging alone would predict. Cumulative inflation-adjusted reimbursement approached one billion reais. Resource use per episode, however, moved in the opposite direction: reimbursement per admission and mean length of stay both declined over the same period. Hospital volume therefore expanded while the mean duration of admission and recorded reimbursement per admission decreased. Statistically significant increases were observed in four of the five macro-regions, with the Northeast being the sole exception.

The increase in hospital admissions occurred against a backdrop of international literature describing a rising incidence of non-melanoma skin cancer over successive decades, with studies of Brazilian populations reporting high rates [3,16]. The Global Burden of Disease Study similarly reported a substantial global contribution of non-melanoma skin cancer to overall cancer incidence [17], and these estimates are themselves sensitive to registration practices because keratinocyte cancers are frequently under-recorded [3]. A recent analysis of SIH/SUS data also found increasing hospital morbidity associated with malignant skin neoplasms, although it combined melanoma and non-melanoma cancers and did not examine long-term reimbursement or resource use [8]. The persistence of the increase after age standardization indicates that population aging alone does not account for the observed expansion in recorded hospital activity. However, the present ecological data cannot determine whether this reflects rising disease incidence, increased diagnostic activity, changes in referral practices, improved access to hospital-based surgical treatment, changes in administrative coding, or movement of care between outpatient and hospital settings. These findings should not be described as evidence of an expansion in epidemiological burden in the absence of external incidence data, and no causal interpretation of the observed trend is intended.

The simultaneous increase in total reimbursement and decrease in reimbursement per admission is consistent with previous evidence that the health-system burden of non-melanoma skin cancer is strongly influenced by treatment volume. In São Paulo, aggregate public-system expenditure for non-melanoma skin cancer exceeded that for melanoma despite a lower estimated cost per treated case [7]. National analyses from Germany and Portugal similarly found that non-melanoma skin cancer generated more episodes and greater aggregate hospital expenditure than melanoma, while expenditure per episode was lower [4,5]. US studies have also documented increasing skin cancer treatment expenditure as the treated population expanded [6,18]. These comparisons should be interpreted cautiously: the present study reports SIH/SUS-approved reimbursement, not the actual resource costs incurred by providers, total health-system expenditure, or economic burden, and the cited studies vary in how costs or expenditures were defined and measured. The observed decline in real reimbursement per admission may reflect several mechanisms that are not mutually exclusive and are specific to the Brazilian reimbursement system: changes to SUS tariff tables (Tabela SUS) and AIH reimbursement values over the study period, shifts in the procedure or case mix billed under ICD-10 C44, changes in procedure-coding practices, and the adequacy of the applied inflation adjustment in tracking the real cost of hospital-based dermatological and surgical care. Because total reimbursement rose despite a sustained decline in reimbursement per admission, growth in admission volume evidently outweighed this per-episode decline. The present findings extend earlier observations by demonstrating this pattern directly, although they cannot distinguish among its underlying mechanisms.

Mean length of stay fell to below one day nationally. One plausible but untested explanation is the increasing use of same-day or short-stay procedural care; the aggregated SIH/SUS data used in this study do not identify the procedures performed and do not permit a reliable distinction between conventional admissions and brief procedural episodes. Physician billing data from Saskatchewan show that most non-melanoma skin cancer treatment occurs in ambulatory settings and across several specialties [19], which is compatible with, but does not confirm, a similar reorganization of care in Brazil. Procedure-level clinical data would be required to distinguish a genuine transition toward same-day surgical management from administrative or billing changes that shorten the recorded episode without a corresponding change in the clinical pathway. The reduction in reimbursement per admission could also reflect erosion of the real value paid by SUS rather than lower clinical complexity, and the two explanations are not mutually exclusive.

Regional differences were substantial, with the South recording the highest age-standardized admission rate and the North the lowest. Previous Brazilian research identified geographic variation in hospital morbidity associated with malignant skin neoplasms and reported higher rates among older individuals and White populations in the source population studied [8]. This association should be treated as an untested hypothesis in the present ecological analysis rather than as an explanation supported by our regional data, which do not include information on skin phenotype. Population-level differences in skin phenotype, cumulative ultraviolet exposure, age structure, and private-sector treatment use are similarly untested hypotheses. Consideration should also be given to differences in access to diagnosis and hospital-based surgical treatment, referral capacity, the balance of public and private healthcare coverage across regions, and the administrative capture of admissions by the SIH/SUS system, all of which are plausible but untested contributors to the observed regional pattern. SIH/SUS data cannot distinguish geographic differences in disease occurrence from differences in access to hospital treatment; a lower recorded rate can therefore reflect a lower disease burden, barriers to diagnosis or referral, or greater reliance on care not captured by SIH/SUS.

The absence of a statistically detectable temporal trend in the Northeast requires caution. The estimate was compatible with a wide range of underlying trajectories, and the absence of a statistically detectable log-linear trend does not establish equivalence or true epidemiological stability. Linkage of hospital data with population-based cancer registries, outpatient procedure records, private-sector data, and measures of regional service supply would be required to determine whether this pattern reflects a distinct epidemiological trajectory or differential access to care.

The marked reduction in the age-standardized admission rate in 2020 was followed by recovery to the highest rate in the series in 2023. A Brazilian SIH/SUS study also reported a reduction in hospitalizations for malignant skin neoplasms during 2020 [20]. This observation is compatible with a temporary disruption of elective and specialized care during the COVID-19 pandemic. Because the present study did not estimate a pandemic effect using interrupted time-series methods, the 2020 reduction should be interpreted descriptively within the longer trend. Re-estimating the national and regional trends after excluding the pandemic period yielded estimates materially similar to the full-period results in direction, magnitude, and statistical significance at the national level and in most macro-regions (Table 5); the pandemic-period disruption therefore does not appear to have materially altered the estimated long-term trend.

The findings have implications for service planning. Shorter stays and lower reimbursement per admission do not imply that aggregate demand is contained: total reimbursement rose sharply as admission volume increased. Planning should account for the cumulative effect of increasing numbers of hospital episodes and marked regional variation. As a general policy implication rather than a conclusion demonstrated by the present analysis, prevention and earlier treatment are plausible strategies for reducing the number of lesions requiring complex hospital care; this study did not evaluate the effectiveness of any prevention program, nor can it determine which specific admissions were clinically avoidable.

This study has several limitations. SIH/SUS includes admissions reimbursed by the public system and excludes most outpatient treatment, all care financed exclusively by private sources, and procedures that do not generate a hospital admission authorization; the estimates therefore represent the publicly financed hospital component of care rather than incidence, prevalence, total treatment expenditure, or societal cost. Admissions represent episodes rather than unique patients and are assigned by place of hospitalization rather than patient residence; therefore, readmissions cannot be identified, and the aggregated data used do not permit the identification or deduplication of interhospital transfers, repeat admissions, or multiple admission authorizations relating to the same tumor. ICD-10 C44 does not distinguish histological subtypes, and diagnoses cannot be validated against pathology; no information on procedure type, disease stage, histology, comorbidity, or case complexity was available. Recorded reimbursement represents approved SUS payment rather than the full economic cost of treatment, and changes in AIH coding practices and SUS reimbursement policy over the study period cannot be excluded as contributors to the observed trends, nor can the gradual migration of care between outpatient, day-hospital, and conventional inpatient settings. Annual population denominators are subject to the uncertainty inherent in population projections. The ecological design precludes inference about individual-level risk factors or causal mechanisms. Each national or regional trend was estimated from only 16 annual observations, limiting statistical power and the precision of the reported estimates, particularly for regional and stratified comparisons. The pooled interaction model assumed independent region-year observations and did not incorporate a region-specific autocorrelation structure and may therefore underestimate the true uncertainty of the regional heterogeneity test; per-region trend estimates, each independently corrected for first-order autocorrelation, remain the primary basis for regional comparison. The study began in 2008 because the selected age-stratified SIH/SUS tabulation was available from that year.

## Conclusions

Recorded SUS-funded hospital activity associated with ICD-10 C44 increased substantially in Brazil between 2008 and 2023, with marked regional variation. Total approved reimbursement increased despite declining real reimbursement per admission and shorter recorded stays. These findings describe trends in publicly financed hospital admission episodes and should not be interpreted as trends in disease incidence, the number of unique patients, or total treatment costs. Integration of hospital, outpatient, cancer registry, and private-sector data would be required to establish how this expansion relates to disease occurrence, treatment pathways, and access to care.

## Appendices

Appendix 1 

**Table 6:** Standard population and age-specific weights used for direct age standardization, Brazil, 2022. The standard population was derived from the 2022 Brazilian Demographic Census (IBGE, SIDRA Table 9514) by five-year age group. Weight indicates the proportion of the total standard population represented by each age group. The 80+ category aggregates the 80-84, 85-89, 90-94, 95-99, and 100-years-or-over groups, as tabulated by IBGE. These weights were applied uniformly to all macro-regions and years in the direct age standardization of the admission rates reported in Table 1.

Age group	Standard population (2022 Census)	Weight
0-4	12,704,860	0.062561
5-9	13,749,440	0.067704
10-14	13,674,961	0.067338
15-19	14,375,942	0.070789
20-24	15,466,463	0.076159
25-29	15,469,723	0.076175
30-34	15,473,117	0.076192
35-39	16,172,791	0.079637
40-44	16,072,170	0.079142
45-49	13,640,112	0.067166
50-54	12,598,581	0.062037
55-59	11,569,106	0.056968
60-64	9,944,389	0.048968
65-69	7,876,232	0.038784
70-74	5,858,536	0.028848
75-79	3,847,379	0.018945
80+	4,586,954	0.022587
Total	203,080,756	1.000000

Appendix 2 

**Table 7:** Annual number of hospital admissions, crude admission rate, and age-standardized admission rate associated with ICD-10 C44 in Brazil, 2008-2023. Admissions (n) are annual counts of hospital admissions with a principal diagnosis of ICD-10 C44 recorded in SIH/SUS. The crude rate was calculated as annual admissions divided by the corresponding total population and multiplied by 100,000. The age-standardized rate was calculated using the direct method, with the age distribution of the 2022 Brazilian Census as the standard population (Appendix 1), and is expressed per 100,000 population.

Year	Admissions (n)	Crude rate per 100,000	Age-standardized rate per 100,000
2008	13,974	7.37	10.43
2009	16,020	8.37	11.79
2010	18,930	9.71	13.19
2011	21,104	10.73	14.31
2012	22,786	11.49	15.01
2013	25,569	12.78	16.35
2014	28,591	14.17	17.75
2015	31,755	15.61	19.09
2016	34,227	16.68	19.97
2017	39,116	18.91	22.16
2018	44,096	21.15	24.18
2019	49,697	23.65	26.39
2020	36,048	17.02	18.53
2021	42,028	19.7	20.92
2022	51,734	24.08	24.94
2023	60,075	27.78	28.03

Appendix 3 

**Table 8:** Autocorrelation coefficient and Durbin-Watson statistic for temporal trend models of hospital admissions and reimbursement associated with ICD-10 C44 in Brazil and its macro-regions, 2008-2023. Rho (ρ) indicates the first-order autocorrelation coefficient estimated and applied in the Prais-Winsten correction for each outcome. The Durbin-Watson statistic was calculated from the ordinary least-squares residuals before this correction and is reported as a diagnostic of serial correlation in the uncorrected series. Values substantially below 2 are consistent with positive autocorrelation and support the use of Prais-Winsten regression rather than ordinary least-squares regression for trend estimation. Outcomes correspond to those reported in Tables 1-4.

Outcome	Region	ρ	Durbin-Watson statistic
Age-standardised admission rate	Brazil	0.433	1.086
Age-standardised admission rate	North	0.328	1.341
Age-standardised admission rate	Northeast	0.552	0.803
Age-standardised admission rate	Southeast	0.667	0.657
Age-standardised admission rate	South	0.442	1.028
Age-standardised admission rate	Central-West	0.156	1.633
Total reimbursement	Brazil	0.412	1.151
Total reimbursement	North	0.487	1.012
Total reimbursement	Northeast	0.426	1.142
Total reimbursement	Southeast	0.711	0.6
Total reimbursement	South	0.584	0.815
Total reimbursement	Central-West	0.497	0.997
Reimbursement per admission	Brazil	0.131	1.661
Reimbursement per admission	North	0.586	0.803
Reimbursement per admission	Northeast	0.426	1.127
Reimbursement per admission	Southeast	0.257	1.4
Reimbursement per admission	South	0.515	1.011
Reimbursement per admission	Central-West	0.453	1.094
Mean length of stay	Brazil	0.862	0.391
Mean length of stay	North	0.8	0.45
Mean length of stay	Northeast	0.827	0.437
Mean length of stay	Southeast	0.898	0.317
Mean length of stay	South	0.611	0.778
Mean length of stay	Central-West	0.299	1.294
